# Estimating direct and indirect costs of hospitalised typhoid fever in Indonesia: A multisite healthcare facility and community survey

**DOI:** 10.1371/journal.pone.0360948

**Published:** 2026-10-26

**Authors:** Jarir At Thobari, Ida Safitri Laksono, Jonathan Hasian Haposan, Hardini Tri Indarti, Asal Wahyuni Erlin Mulyadi, Alindina Anjani, Emma Watts, Julie Bines

**Affiliations:** 1 Center for Child Health, Faculty of Medicine, Public Health and Nursing, Universitas Gadjah Mada, Yogyakarta, Indonesia; 2 Department of Pharmacology and Therapy, Faculty of Medicine, Public Health and Nursing, Universitas Gadjah Mada, Yogyakarta, Indonesia; 3 Department of Child Health, Faculty of Medicine, Public Health and Nursing, Dr. Sardjito General Hospital, Universitas Gadjah Mada, Yogyakarta, Indonesia; 4 Enteric Diseases Research Group, Murdoch Children’s Research Institute, Parkville, Australia; 5 Department of Biostatistics, Epidemiology, and Population Health, Faculty of Medicine, Public Health and Nursing, Universitas Gadjah Mada, Yogyakarta, Indonesia; 6 Department of Paediatrics, the University of Melbourne, Parkville, Australia; 7 STIKes Budi Luhur Cimahi, West Java, Indonesia; 8 Faculty of Social and Political Sciences, Universitas Sebelas Maret Surakarta, Central Java, Indonesia; 9 Department of Internal Medicine, Faculty of Medicine, Public Health and Nursing, Universitas Gadjah Mada, Yogyakarta, Indonesia; 10 Department of Gastroenterology and Clinical Nutrition, the Royal Children’s Hospital, Parkville, Australia; Universitas Padjadjaran, INDONESIA

## Abstract

**Background:**

Typhoid fever, caused by the bacterium *Salmonella enterica* serovar Typhi (*S.* Typhi), remains a major global threat despite substantial declines over the past three decades. In 2021, an estimated 7.1 million (5.6 to 9.2 million) cases occurred worldwide. Following the WHO’s recommendation, ten countries (excluding Indonesia) have introduced the typhoid conjugate vaccine (TCV). To inform decision-making for the potential introduction of TCV into Indonesia’s National Immunisation Program, we estimated the direct and indirect costs of typhoid fever in Indonesia.

**Methods:**

We conducted a multisite, incidence-based cost-of-illness study using a patient-level bottom-up costing approach in 23 hospitals and primary health centres (PHCs) across six provinces. Children (6 months to 17 years) and adults (≥18 years) hospitalised with ICD-10 A01 typhoid fever hospitalised with at least one positive typhoid serodiagnostic result during 2019-2023 (retrospective) and 2022-2023 (prospective), without other major infectious or chronic comorbidities, were identified from facility records. Direct medical costs were obtained retrospectively from hospital/PHC bills and clinical records, while direct non-medical and indirect (productivity loss) costs were collected prospectively through interviewer-administered household questionnaires. Sensitivity analyses re-estimated costs after excluding component-wise outliers and imputing missing income values using the provincial minimum wage.

**Results:**

We collected 2,069 records (1,538 retrospective [891 children; 647 adults] and 531 prospective [329 children; 202 adults]) across six provinces. The weighted mean direct medical costs were US$177.0 ± 101.9 for children and US$199.8 ± 119.8 for adults, while the corresponding direct non-medical costs were US$43.7 ± 16.3 and US$39.0 ± 10.9, respectively. Indirect costs were US$23.9. ± 5.6 (children) and US$17.9 ± 5.5 (adults). Private hospitals had the highest total direct medical cost (US$254.4), while PHCs had the lowest (US$47.8). Costs varied substantially by provinces (BPJS tariff regions).

**Conclusion:**

Hospitalised typhoid fever imposes a substantial economic burden in Indonesia. These setting- and region-specific cost estimates can inform cost projections and cost-effectiveness analyses for the introduction of the typhoid conjugate vaccine in Indonesia.

## Introduction

Typhoid fever is a systemic infection caused by the bacterium *Salmonella enterica* serovar Typhi (*S.* Typhi) and transmitted predominantly via the faecal-oral route. Typhoid fever remains a substantial health challenge in many low- and middle-income countries (LMICs) [1,2]. Global trends indicate a significant decline in the incidence of typhoid fever over the last three decades, which is associated with improvements in water, sanitation, and hygiene (WASH), food safety, and economic development [3,4]. In 2021, typhoid was estimated to be responsible for 7.1 million (5.6 to 9.2 million) cases and 93,000 (46,000–163,000) deaths globally, with the highest incidence in South Asia, South-East Asia, Oceania, and southern Sub-Saharan Africa [3]. When left untreated, typhoid fever can result in serious complications in 10–15% of patients [5]. The emergence of antimicrobial-resistant and extensively drug-resistant strains of *S.* Typhi has greatly complicated treatment and preventive strategies [6]. The World Health Organization has recommended the introduction of Typhoid Conjugate Vaccine (TCV) into national immunisation programs in countries where typhoid is endemic [5,7–9].

As the world’s fourth most populous country, Indonesia had a population of 284.5 million in 2025 and is considered endemic for typhoid fever [3,10]. In 2021 alone, an estimated 470 thousand total typhoid cases were reported (168 cases per 100,000 population) [11]. Although empirical data on typhoid hospitalisation in Indonesia are limited, a modelling study estimated that, in the absence of TCV immunisation or other interventions, approximately 120 thousand hospitalisations and 70,800 deaths due to typhoid would occur in Indonesia between 2025 and 2054 [12]. Introduction of TCV into the National Immunisation Program in Indonesia may offer significant benefits for individuals, their families and the healthcare system. In 2002, a cost-of-illness study conducted in five countries, including Indonesia, estimated that hospitalisation costs in Indonesia were US$884 per typhoid case [13,14]. However, this healthcare cost estimate was based on data from only two districts in North Jakarta, limiting its representativeness across regions and provinces in Indonesia. This study was also conducted before the implementation of the national health insurance program (’Jaminan Kesehatan Nasional’). Jaminan Kesehatan Nasional (JKN) is an Indonesian National Health Insurance program available to all Indonesian citizens since 2014, managed by the Indonesian Social Security Agency on Health (’Badan Penyelenggara Jaminan Sosial Kesehatan’ or BPJS) [15,16]. This evidence gap highlights the need for comprehensive, up-to-date cost-of-illness data to refine economic inputs and improve the accuracy of cost-effectiveness analyses.

Therefore, this study aimed to estimate the direct and indirect costs associated with hospitalisation for probable typhoid cases in both children and adults, across various healthcare settings and regions in Indonesia. This data will be essential for informing policymakers on the cost-effectiveness of adding TCV to the national immunisation program across diverse regions of Indonesia.

## Materials and methods

### Study design and sampling method

This study employed a multisite, incidence-based, cost-of-illness evaluation using a patient-level bottom-up costing approach, which utilised both retrospective and prospective surveys in healthcare facilities and community households across Indonesia [17]. In this study, we focused on hospitalised cases only because inpatient treatment represents a resource-intensive component of typhoid care and is an important input for economic evaluation of interventions that prevent severe disease and hospitalisation. The study adopted a societal perspective by incorporating direct medical costs, direct non-medical costs, and indirect costs of typhoid fever hospitalisations. These cost components were selected to inform a subsequent cost-effectiveness and budget impact analysis of introducing TCV into Indonesia’s national immunisation program, which was recently published [12]. The study surveyed 12 public hospitals (tertiary and secondary), 6 private hospitals, and 5 primary health centres (PHCs) across 6 provinces in Indonesia. The selection flow of study sites and patients is described in Fig 1.

**Fig 1:**
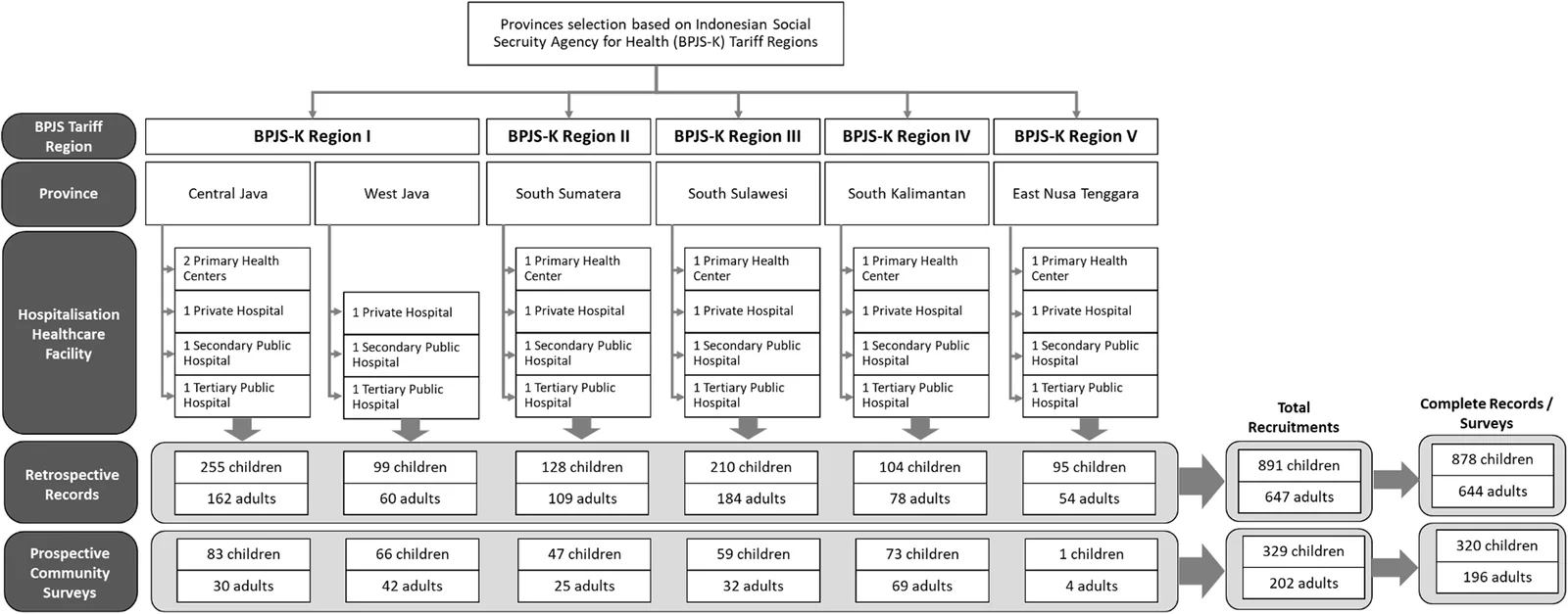
Flow chart of overall study recruitment and data collection.

The study provinces and facilities were purposively selected to capture heterogeneity in typhoid burden, geographical setting, facility ownership, level of care, and the Indonesian Social Security Agency for Health (BPJS) tariff-region classification. BPJS categorises all provinces in Indonesia into five-tier tariff regions (I-V) based on the similarity of consumer price indices [18]. The selected provinces would represent other provinces within the same BPJS tariff regions and reflect the national variations in healthcare costs across Indonesia. Within each province, facilities were selected to represent different levels, sizes, and types of care, including the secondary and tertiary public hospitals, private hospitals, and PHCs. Facility willingness to participate and feasibility were also considered for the selection criteria. This sampling strategy was intended to achieve the multi-region and a range of healthcare settings for further economic evaluations [18], including cost-effectiveness and budget impact analyses.

The INA-CBG (Indonesian Case Based Groups) tariff was not applied directly as the cost of hospitalisation, because INA-CBG represents a prospective case-based reimbursement amount and may differ from the expenditures recorded by a facility for the services delivered to an individual patient [19]. Differences between INA-CBG reimbursement and hospitalisation costs have also been reported empirically in Indonesia [20–22]. Therefore, BPJS tariff-region classification was used for geographical stratification of study sites, but a bottom-up patient-level costing approach based on itemised individual billing and health records was used.

### Participants and case definition

The study population comprised children (aged 6 months to 17 years) and adults (aged 18 years and above) hospitalised with a diagnosis of typhoid fever (ICD-10-CM code A01) at the designated study facilities. Although typhoid incidence is generally higher among older children, children aged 6 months and above were included to ensure that the study population was relevant to subsequent economic evaluations of TCV introduction, as the WHO recommends TCV use from 6 months of age in typhoid-endemic settings [7,8]. Eligibility also required a documented positive typhoid serodiagnostic result obtained, including Widal, TUBEX TF, Typhidot, or a typhoid-specific IgM/IgG antibody test. As culture confirmation was not required and not a routine test in Indonesia [23], all included participants were classified as probable typhoid cases, rather than culture-confirmed cases [9,24,25]. Individuals with other concurrent infectious diseases (such as COVID-19, pneumonia, dengue, malaria, etc.) and comorbid conditions during hospitalisation (including but not limited to diabetes, cardiovascular diseases, asthma, cancers, autoimmune diseases, HIV, etc.) were excluded. This study utilised retrospective (for direct medical costs) and prospective data collection (for direct non-medical and indirect costs). Prospective participants were recruited between 23/09/2022 and 20/10/2023. For the retrospective study, health records and billings were accessed between 20/09/2022 and 15/03/2023, covering inpatient admissions from 01/01/2019 to 21/02/2023. All investigators and research assistants involved in the study received training in Good Clinical Practice (GCP) principles, data entry and management procedures, and interviewing techniques, as applicable to their roles, prior to study conduct.

### Inpatient direct medical costs

Direct medical costs are expenses directly associated with the provision of medical care, which may be reimbursed to the facility through insurance or paid out of pocket. These costs were retrospectively collected from hospital and PHC billing data, using either paper-based (primarily in primary health centres and hospitals in rural and suburban areas) or electronic formats (primarily in tertiary hospitals and cities). The costing and billing data were acquired from the accounts maintained by the finance department of hospitals and PHCs. All cost variables were documented according to the individual patient billing records at the time of hospitalisation. Additionally, demographic information, including age, gender, hospitalisation and discharge dates, as well as clinical and laboratory data, were obtained.

Various cost categories, including administration costs, room charges, professional medical fees, diagnostic test expenses, and medication costs, were collected to determine the direct medical expenses associated with typhoid fever treatment. Administration costs were defined as the aggregate expenses levied for registration and other administrative procedures. Room charges encompassed all expenses incurred for occupying a designated room, depending on the selected hospital class. These charges included all associated costs for bed utilisation, water services, electricity, and meals, whether in a general room or an intensive care unit setting. Professional medical fees comprise the overall expenses attributed to doctor and nurse services rendered during a patient’s treatment. Diagnostic costs include expenses for laboratory tests and radiology examinations performed on patients. Medication costs comprised expenses incurred for all medications administered to patients during hospitalisation, as well as the utilisation of medical consumables throughout the course of treatment.

### Direct non-medical

Direct non-medical costs refer to expenses incurred by patients and their families associated with treatment that are not directly related to medical care. Indirect costs, on the other hand, pertain to productivity losses resulting from illness. To estimate household direct non-medical and indirect costs per hospitalised case of typhoid fever, face-to-face interviews were conducted with patients or their caregivers. Data were collected using a self-reported questionnaire containing all illness-related expenditures from symptom onset to recovery at home after discharge.

Direct non-medical costs include transportation expenses, complementary therapy, and food supplements. Complementary therapy included all therapies and medications used for the treatment of typhoid fever, purchased without a prescription, such as over-the-counter medications (antipyretic drugs), supplemental vitamins, and herbal remedies. Food supplements comprise food items purchased by the patient or caregiver that are intended to aid recovery, such as protein-rich foods, rehydration drinks, and yoghurt. Transportation costs include expenses for transporting the patient and their parent/guardian to and from the hospital.

### Indirect costs

This study used productivity loss during hospitalisation as the main indirect cost component, while productivity losses from premature mortality and outside the hospitalisation period were not included. The income loss was used as a proxy for productivity loss using a wage-based human-capital approach [26]. Self-reported monthly income, collected through the household questionnaire, was used to approximate the economic value of productive time lost from work due to accessing healthcare or being hospitalised or caring for the hospitalised patient (as a parent/guardian/caregiver). The patient or caregiver’s monthly income was converted into an hourly income rate, assuming the standard full-time employment of a 40-hour, five-day working week under Indonesian labour regulations [27]. The productivity loss was calculated by multiplying the hourly income rate by the average number of work hours absent during the patient’s hospitalisation.

### Healthcare facility-specific cost weighting method

To reduce bias from non-random distribution and account for differences in the distribution of hospitalised patients across health facility types, direct medical, direct non-medical, and indirect costs were weighted according to the estimated proportion of patients hospitalised in each facility type. The weights were derived from 1% sample data of the BPJS claims between 2015 and 2020 across all facility types, including private hospitals. The sample dataset comprised 2.2 million insured participants and 28.1 million claim records [28,29]. Up to 2020, BPJS insurance covered approximately 82.3% of Indonesia’s total eligible population [30]. The dataset was used to estimate, within each age group, the proportion of typhoid fever treated in four facility categories: tertiary (class A/B) public hospitals, secondary (class C/D) public hospitals, private hospitals, and primary health centres. Overall, 7.8% of cases were managed in tertiary public hospitals, 13.9% in secondary public hospitals, 49.8% in private hospitals, and 28.4% in primary health centres. These proportions were applied as weights to the facility-specific cost estimates from the costing study results to generate weighted average costs. The weighted average cost was calculated as the sum of each facility-specific mean multiplied by the corresponding facility weight. A similar cost-weighting approach has been applied in a costing study of childhood diarrhea in Indonesia [18].

### Data management and analysis

All data were collected and entered into electronic case report forms (eCRFs) using the REDCap® web-based data management platform by research assistants at each study site, and were managed by a data manager at the Center for Child Health, Universitas Gadjah Mada. Statistical analysis was conducted using Stata Statistical Software, version 15, for Windows (StataCorp, College Station, TX) [31]. All monetary values were converted from Indonesian rupiah (IDR) to 2023 US dollars (USD), using the exchange rate of 1 US$ = 15,000 IDR. Given the relatively short data-collection period for the household survey (2022–2023), no additional inflation adjustment was applied. For consistency and simplicity, historical facility cost data from 2019 to 2023 were also converted using the same approach and were not separately adjusted for inflation. Numerical data are expressed as mean ± standard deviation (SD). Descriptive statistics were used to present most data as absolute numbers, percentages, or mean ± SD. Supplementary tables provide additional details, including the median, the interquartile range, and minimum and maximum values.

### Sensitivity analysis

To test the robustness of cost estimates to extreme values, a sensitivity analysis was conducted. We re-analysed all cost estimates after excluding outliers (those below or above the 3rd quartile plus 1.5 times the interquartile range) within each cost component stratum. Outliers were analysed and flagged component-wise, so that exclusions applied only to that component. For the sensitivity analysis of indirect costs, missing income values were imputed using the officially published provincial minimum wage rates in Indonesia [32].

### Ethical considerations

The study received approval from the Medical and Research Ethics Committee of the Faculty of Medicine, Public Health, and Nursing Universitas Gadjah Mada, Indonesia (Approval No. KE/FK/1103/EC/2022) as well as from the Royal Children’s Hospital Melbourne Human Research Ethics Committee, Australia (Reference Number 88511/RCHM-2022). Data collection commenced after ethics committee approval, including approval of all subsequent protocol amendments. Written informed consent was obtained from all participants prior to enrolment in the prospective community surveys; for minors, consent was obtained from a parent or legal guardian (and assent from the child when applicable).

## Results

This study analysed data from 2,069 records (1,538 retrospective [891 children; 647 adults] and 531 prospective [329 children; 202 adults]) who were hospitalised with typhoid fever at the respective sites. The distributions of these populations by region, health facility, and source are shown in Fig 1, Table 1, and S1-S2 Tables in S1 File. All retrospective records and prospective participants documented at least one positive typhoid serodiagnostic test result. Among retrospective records, the sole widal test was the predominant serodiagnostic test, accounting for 69% of positive results among children and 74% among adults (S3 Table in S1 File). Among collected health records, 62.3% adults were female; 8.7% experienced complications, and 96.6% were discharged recovered. Educational attainment was predominantly senior high school (54.6%), and 33.7% were unemployed.

**Table 1:** Participant characteristics in retrospective records (child and adult groups).

*Parameter*	Public Hospitals		Private Hospitals	Primary Health Centres	Total
	Tertiary	Secondary			
	(Class A/B)	(Class C/D)			
***RETROSPECTIVE RECORDS***					
**No. of records (n)**	307	525	519	187	1538
**No. by BPJS regions, n (%)**					
Region I	105 (34.2)	204 (38.9)	234 (45.1)	124 (66.3)	667 (43.4)
Region II	53 (17.3)	142 (27.0)	85 (16.4)	0 (0.0)	280 (18.2)
Region III	42 (13.7)	70 (13.3)	70 (13.5)	35 (18.7)	217 (14.1)
Region IV	80 (26.0)	85 (16.2)	122 (23.5)	28 (15.0)	315 (20.5)
Region V	27 (8.8)	24 (4.6)	8 (1.5)	0 (0.0)	59 (3.8)
**Sex, n (%)**					
Male	146 (47.6)	222 (42.3)	226 (43.5)	68 (36.4)	662 (43.0)
Female	161 (52.4)	303 (57.7)	293 (56.5)	119 (63.6)	876 (57.0)
**Age groups**					
*Child group., mean ± SD in y.o*	*8.0 ± 4.6*	*8.4 ± 4.8*	*8.0 ± 4.7*	*9.1 ± 5.0*	*8.3 ± 4.8*
1-5 y.o.*, n (%)*	67 (34.2)	96 (35.0)	112 (36.8)	34 (29.1)	309 (34.7)
6-12 y.o.*, n (%)*	95 (48.5)	112 (40.9)	129 (42.4)	45 (38.5)	381 (42.8)
13-17 y.o.*, n (%)*	33 (16.8)	65 (23.7)	59 (19.4)	36 (30.8)	193 (21.7)
*Adult group., mean ± SD in y.o*	*32.3 ± 13.9*	*33.8 ± 13.9*	*32.4 ± 12.9*	*28.5 ± 11.3*	*32.5 ± 13.4*
18-25 y.o.*, n (%)*	47 (42.3)	94 (37.5)	83 (38.6)	36 (51.4)	260 (40.2)
26-55 y.o.*, n (%)*	51 (45.9)	132 (52.6)	117 (54.4)	33 (47.1)	333 (51.5)
>55 y.o.*, n (%)*	11 (9.9)	24 (0.4)	15 (6.9)	1 (1.4)	51 (7.9)
**Payment type, n (%)**					
Out of pocket	38 (12.4)	41 (7.8)	73 (14.1)	53 (28.3)	205 (13.3)
JKN insurance	269 (87.6)	483 (92.0)	409 (78.8)	134 (71.7)	1295 (84.2)
Private insurance	0 (0.00)	1 (0.2)	37 (7.1)	0 (0.0)	38 (2.5)
**Type of Typhoid, n (%)**					
Complicated	13 (4.2)	26 (4.9)	65 (12.5)	1 (0.5)	105 (6.8)
Uncomplicated	294 (95.8)	499 (95.1)	454 (87.5)	186 (99.5)	1433 (93.2)
**Outcome: n (%)**					
Recovered	301 (98.1)	496 (94.5)	512 (98.6)	180 (96.3)	1489 (96.8)
Died	1 (0.3)	1 (0.2)	1 (0.2)	0 (0.0)	3 (0.2)
Discharged against medical advice	5 (1.6)	27 (5.1)	6 (1.2)	3 (1.6)	41 (2.7)
Referred	0 (0.0)	1 (0.2)	0 (0.0)	4 (2.1)	5 (0.3)

Note: Missing data are not shown in the table. Percentages were calculated using the total sample size, including observations with missing values.

The most frequently reported household characteristics were a monthly income of above US$240 (31.8%) and a household size of 4 (31.1%). Nearly all adult patients were accompanied by a caregiver during hospitalisation (185/196; 94.4%). Most facility charges (hospital or primary health centre) were covered by JKN, private insurance, or other third parties (86.7%) (Table 1). Despite this coverage, 4.6% adult and 12.5% child inpatients reported making partial out-of-pocket payments. Among these participants, 71.4% adult and 71.8% child inpatients reported that the payments were mainly for room upgrades. Most adults (83.7%) and children (85.6%) received care at facilities located >1 km from their residence, and motorbikes were the most common mode of transport (37.2% adults and 42.3% children). Beyond facility-based care, 28.6% of adults and 28.7% of children reported using complementary and alternative medicine, most commonly herbal/traditional remedies (41.0% adult users and 35.9% child users). Separately, 28.6% of adults and 40.0% of children reported using dietary supplements, most often high-protein foods (39.0% adult users and 34.4% child users).

Among children, 53.0% were male; 5.5% experienced complications, and 97.0% were discharged recovered. Parents were the primary caregivers (84.7%). More than 30% of parents reported having completed senior high school or higher education (including university). Fathers were most commonly employed in the private sector (29.2%), whereas over half of mothers were unemployed (51.4%). Most facility charges were covered by insurance or other third parties (77.1%); however, 12.5% reported some out-of-pocket payments, mainly for room upgrades (71.8%). The most common household income category was US$120–US$240 per month (37.2%), and the most frequently reported household size was four members (30.9%).

From the community surveys, most respondents were highly educated (above high school level) in both the adult and parent/caregiver groups (62.1%–76.5%). Among adults, 33.7% of respondents were unemployed or students, followed by those in other occupations (e.g., farmers, fishers, labourers, or domestic workers) at 26.0%. In the parent/caregiver group, the largest share worked in other occupations (22.2%), followed by private-sector employees (20.0%) (Table 2).

**Table 2:** Participant characteristics in prospective community surveys (child and adult groups).

*Parameter*	Public Hospitals		Private Hospitals	Primary Health Centres	Total
	Tertiary	Secondary			
	(Class A/B)	(Class C/D)			
***PROSPECTIVE SURVEYS***					
No. of surveys (n)	99	194	188	50	531
**Age of patients by group*, mean ± SD in y.o***					
Child group	7.7 ± 4.4	8.4 ± 5.0	7.8 ± 4.6	9.6 ± 5.2	8.1 ± 4.8
Adult group	28.9 ± 10.3	34.2 ± 14.9	32.2 ± 13.9	29.4 ± 13.2	32.3 ± 13.9
**Education of adult patients, *n(%)***					
Above high school	3 (12.5)	13 (16.5)	22 (30.6)	4 (19.1)	42 (21.4)
High school or lower	21 (87.5)	63 (79.7)	49 (68.1)	17 (80.9)	150 (76.5)
**Occupation of adult patients, *n(%)***					
Government employees	2 (8.3)	14 (17.7)	3 (4.2)	0 (0.00)	19 (9.7)
Private-sector employees	1 (4.2)	14 (17.7)	21 (29.2)	3 (14.3)	39 (19.9)
Entrepreneur	6 (25.0)	9 (11.4)	2 (2.8)	4 (19.1)	21 (10.7)
Other occupations	5 (20.8)	17 (21.5)	20 (27.8)	9 (42.9)	41 (26.0)
Unemployed/students	10 (41.7)	25 (31.7)	26 (36.1)	5 (23.8)	66 (33.7)
**Parents’ demography (for child group only)**					
**Age of parents, *mean ± SD in y.o***					
Mothers	36.9 ± 6.9	36.5 ± 7.3	36.3 ± 7.1	39.2 ± 8.6	36.8 ± 7.3
Fathers	40.5 ± 8.8	39.6 ± 7.9	39.5 ± 7.8	42.9 ± 10.3	40.1 ± 8.4
**Education of parents, *n(%)***					
*Mothers*					
Above high school	31 (43.1)	33 (32.0)	53 (45.7)	1 (3.5)	118 (36.9)
High school or lower	40 (55.5)	69 (66.9)	62 (53.4)	28 (96.5)	119 (62.1)
*Fathers*					
Above high school	27 (37.5)	23 (22.3)	49 (42.2)	2 (6.9)	101 (31.6)
High school or lower	41 (56.9)	74 (71.8)	61 (52.6)	27 (93.1)	203 (63.4)
**Occupation of parents, *n (%)***					
Government employees	7 (9.7)	2 (1.9)	11 (9.5)	1 (3.5)	21 (6.6)
Private-sector employees	11 (15.3)	8 (7.8)	42 (36.2)	3 (10.3)	64 (20.0)
Entrepreneur	15 (20.8)	13 (12.6)	10 (8.6)	4 (13.8)	42 (13.1)
Other occupations	16 (22.2)	34 (33.1)	16 (13.8)	5 (17.2)	71 (22.2)
**Participants / caregivers / family members missed work due to participants’ illness, *n (%)***					
Child group					
Yes	49 (68.1)	57 (55.3)	79 (68.1)	13 (44.8)	198 (61.9)
No	23 (31.9)	46 (44.7)	37 (31.9)	16 (44.2)	122 (38.1)
Adult group					
Yes	14 (58.3)	49 (62.0)	34 (47.2)	14 (66.7)	111 (56.6)
No	10 (41.7)	30 (37.9)	38 (52.8)	7 (33.3)	85 (43.4)

Note: Missing data are not shown in the table. Percentages were calculated using the total sample size, including observations with missing values.

### Direct medical costs

Across all facility types, direct medical costs were the largest component for both children and adults, with the highest costs in private hospitals and the lowest in primary health centres (PHCs). Among children, the unweighted mean direct medical cost per hospitalised typhoid case ranged from US$157.4 ± 86.1 in secondary public hospitals up to US$254.4 ± 176.4 in private hospitals, compared with US$47.8 ± 21.8 in PHCs, where costs were recorded as a package cost. Furthermore, the unweighted mean direct medical cost per case among adults ranged from US$177.3 ± 101.8 in tertiary public hospitals to US$290.1 ± 28.7 in private hospitals, compared with US$46.6 ± 28.7 in PHCs as a package cost. After applying weighting based on national-level healthcare-seeking patterns, the weighted mean direct medical costs of typhoid fever were estimated at US$177.0 ± 101.9 in children and US$199.8 ± 119.8 in adults (Table 3).

**Table 3:** Direct medical cost, direct non-medical cost and indirect cost of hospitalised typhoid fever by healthcare facility type and age groups.

Parameter	Children (6 m.o. to 17 y.o.)					Adults (≥18 y.o.)				
	No. of non-missing records	Public Hospitals		Private Hospitals		No. of non-missing records	Public Hospitals		Private Hospitals	Primary Health Centres
		Tertiary	Secondary		Primary Health Centres		Tertiary	Secondary		
		(Class A/B)	(Class C/D)				(Class A/B)	(Class C/D)		
**No. of complete records**	878	195	274	304	105	644	111	251	215	67
**Care-seeking distribution (weight) across facilities,** *% of all cases*		7.8%	13.9%	49.9%	28.4%		7.8%	13.9%	49.9%	28.4%
**Length-of-stay in wards,** *Mean* ± SD in days	889	4.0 ± 2.8	3.8 ± 1.3	3.5 ± 1.7	2.9 ± 1.1	647	3.7 ± 1.8	3.7 ± 1.6	3.5 ± 1.6	2.9 ± 0.8
*Weighted length-of-stay*		*3.4 ± 0.4*					*3.4 ± 0.4*			
**Direct Medical Cost,** *Mean* ± SD in USD										
Administrative Cost	661	3.7 ± 4.7	5.8 ± 7.4	7.6 ± 4.5	NA	507	3.5 ± 5.2	5.3 ± 6.8	7.7 ± 4.2	NA
Room cost	771	50.8 ± 35.2	51.6 ± 42.7	86.7 ± 77.3	NA	573	52.2 ± 53.7	56.7 ± 52.3	86.4 ± 81.3	NA
Professional Fee	771	75.2 ± 46.9	54.1 ± 33.3	72.5 ± 58.9	NA	572	60.5 ± 25.7	64.2 ± 44.9	68.6 ± 51.5	NA
Medication cost	771	25.9 ± 32.3	27.0 ± 19.9	55.9 ± 59.1	NA	576	27.7 ± 25.5	47.9 ± 48.9	80.9 ± 86.7	NA
Diagnostic Cost	733	36.6 ± 46.7	21.7 ± 12.1	32.3 ± 27.2	NA	567	38.1 ± 43.3	29.9 ± 16.9	46.9 ± 36.4	NA
*Unweighted total direct medical Cost*	*(N=878)*	*187.4 ± 114.2*	*157.4 ± 86.1*	*254.4 ± 176.4*	47.8 ± 21.8*	*(N=644)*	*177.3 ± 101.8*	*201.0 ± 107.8*	*290.1 ± 187.9*	46.6 ± 28.7*
*Weighted total direct medical cost*		***177.0 ± 101.9***					***199.8 ± 119.8***			
**Direct Non-Medical Cost,** *Mean* ± SD in USD										
Transportation cost	317	19.6 ± 46.1	16.6 ± 17.2	13.3 ± 20.9	6.1 ± 5.4	172	23.3 ± 55.2	12.0 ± 10.5	13.3 ± 14.3	7.0 ± 6.7
Accommodation cost	28	41.3 ± 96.7	17.9 ± 11.9	33.3 ± 67.2	7.8 ± 5.1	14	7.9 ± 7.7	28.3 ± 2.4	7.9 ± 4.5	2.2 ± 0.8
Meal cost	306	43.5 ± 69.9	33.1 ± 32.1	24.3 ± 21.6	16.5 ± 13.9	163	34.5 ± 31.4	24.9 ± 15.9	22.7 ± 19.1	17.7 ± 9.8
Food supplement cost	128	11.9 ± 10.8	20.8 ± 23.1	5.7 ± 5.4	12.9 ± 13.8	44	30.0 ± 38.3	14.2 ± 13.2	12.8 ± 18.2	13.6 ± 12.7
Complementary therapy cost	88	19.0 ± 41.3	4.2 ± 3.6	5.3 ± 5.4	4.9 ± 3.4	48	8.8 ± 6.7	6.0 ± 8.0	5.0 ± 4.8	2.3 ± 1.1
*Unweighted total direct non-medical cost*	*(N=320)*	*78.2 ± 154.8*	*63.2 ± 53.4*	*40.3 ± 37.6*	*30.7 ± 26.2*	*(N=196)*	*66.4 ± 86.5*	*44.3 ± 28.7*	*37.9 ± 28.8*	*30.9 ± 25.0*
*Weighted total direct non-medical cost*		***43.7 ± 16.3***					***39.0 ± 10.9***			
**No. of complete surveys**	198	49	57	79	13	111	14	49	34	14
**Indirect cost,** *Mean* ± SD in USD										
Income lost among those who missed work due to illness	198	44.3 ± 56.3	35.6 ± 47.1	40.6 ± 31.8	39.2 ± 41.7	111	9.0 ± 13.8	31.2 ± 24.9	45.6 ± 62.7	21.7 ± 25.2
*Unweighted total indirect cost*	*(N=320)*	*30.1 ± 50.7*	*19.7 ± 39.2*	*27.6 ± 32.3*	*17.6 ± 33.7*	*(N=196)*	*5.3 ± 11.4*	*19.4 ± 24.8*	*21.5 ± 48.5*	*14.5 ± 22.9*
*Weighted total indirect cost*		***23.9. ± 5.6***					***17.9 ± 5.5***			

Note: *Direct medical costs for Primary Health Centre (PHC) were recorded as a package without itemised components

The breakdown revealed that administrative costs accounted for 3.3% and 2.8% of total direct medical costs in children and adults, respectively; room costs for 35.3% and 31.7%; professional fees for 36.1% and 30.9%; medication costs for 20.7% and 26.8%; and diagnostic costs for 16.0% and 17.9%. The length of stay of patients also influences direct medical costs. Length of stay (LOS) varied across facility types, with the longest stays in tertiary public hospitals (children: 4.0 ± 2.8 days; adults: 3.7 ± 1.8), followed by secondary public hospitals (3.8 ± 1.3; 3.7 ± 1.6), private hospitals (3.5 ± 1.7; 3.5 ± 1.6), and primary health centres (2.9 ± 1.1; 2.9 ± 0.8). The weighted means of LOS were similar in children and adults (3.4 ± 0.4 days) (Table 3)

Costs varied markedly by provinces (BPJS tariff regions). Among children, the mean direct medical costs were highest in South Sulawesi (US$255.3 ± 154.2) and South Sumatra (US$218.5 ± 91.0), and lowest in Central Java (US$111.2 ± 57.6). In adults, the highest mean direct medical costs were observed in East Nusa Tenggara (US$305.6 ± 226.8) and West Java (US$247.2 ± 231.5), while the lowest costs were found in Central Java (US$168.9 ± 125.9). Provinces with higher direct medical costs tend to have higher direct non-medical costs and indirect costs as well (Table 4).

**Table 4:** Direct medical cost, direct non-medical cost and indirect cost of hospitalised typhoid fever by provinces and age groups.

	Children (6 m.o. to 17 y.o.)								Adults (≥18 y.o.)					
	No. of non-missing data	West Java	Central Java	South Sumatra	South Sulawesi	South Kalimantan	East Nusa Tenggara	No. of non-missing records	West Java	Central Java	South Sumatra	South Sulawesi	South Kalimantan	East Nusa Tenggara
		Region I	Region I	Region II	Region III	Region IV	Region V		Region I	Region I	Region II	Region III	Region IV	Region V
**No. of complete records**	878	181	229	153	127	160	28	644	127	130	127	90	139	31
**Direct Medical Cost,** *Mean* ±SD in USD														
Administrative Cost	661	7.8 ±6.2	2.6 ±1.7	9.8 ±8.1	4.2 ±2.5	7.2 ±2.2	3.1 ±0.6	507	8.5 ±6.4	2.3 ±1.8	9.3 ±7.6	4.5 ±2.4	3.6 ±2.6	5.2 ±5.1
Room cost	771	80.6 ±92.1	45.0 ±26.2	84.0 ±54.8	75.4 ±33.0	44.2 ±53.1	78.5 ±58.7	573	102.9 ±101.5	48.8 ±37.7	77.8 ±52.8	67.2 ±54.5	35.2 ±37.3	83.6 ±76.2
Professional Fee	771	63.3 ±54.9	31.8 ±16.6	66.2 ±39.1	118.8 ±62.3	78.7 ±28.5	37.8 ±23.9	572	61.8 ±57.8	46.1 ±44.0	55.1 ±24.1	83.7 ±54.9	74.1 ±24.4	95.3 ±54.6
Medication cost	771	49.9 ±66.7	34.8 ±22.3	34.7 ±37.4	37.3 ±29.1	37.5 ±51.3	17.2 ±18.1	576	57.3 ±64.4	69.4 ±88.1	71.9 ±58.5	26.5 ±21.3	45.3 ±38.9	65.5 ±127.4
Diagnostic Cost	733	36.5 ±32.2	18.6 ±8.7	24.4 ±12.7	57.6 ±56.3	21.3 ±14.9	31.9 ±19.6	567	49.3 ±46.3	34.6 ±14.8	47.8 ±48.0	30.2 ±12.3	56.6 ±43.6	56.6 ±43.6
*Unweighted total direct medical cost*	*(N=878)*	*207.3 ±212.9*	*111.2 ±57.6*	*218.5 ±91.0*	*255.3 ±154.2*	*177.9 ±116.7*	*167.9 ±84.8*	*(N=644)*	*247.2 ±231.5*	*168.9 ±125.9*	*238.0 ±100.4*	*196.6 ±120.7*	*178.9 ±90.4*	*305.6 ±226.8*
**Direct Non-Medical Cost,** *Mean* ±SD in USD														
Transportation cost	317	14.1 ±18.2	10.4 ±9.6	20.0 ±13.7	22.7 ±52.9	12.3 ±23.5	3.3 ±NA	195	21.7 ±42.3	10.0 ±8.8	21.5 ±13.5	10.5 ±10.1	10.5 ±12.4	12.7 ±13.3
Accommodation cost	28	14.4 ±11.7	10 ±4.7	12.7 ±9.3	41.4 ±86.4	5.7 ±3.3	NA	15	14.1 ±12.1	2.2 ±0.8	62.3 ±NA	10.1 ±10.0	12.5 ±5.9	NA
Meal cost	306	24.2 ±18.2	21.4 ±17.6	36.2 ±28.7	56.2 ±80.1	24.6 ±27.5	27 ±NA	186	31.4 ±26.9	21.7 ±11.7	40.5 ±32.4	29.9 ±27.7	20.5 ±12.7	15.4 ±7.9
Food supplement cost	128	12.9 ±11.4	4.5 ±3.7	24.4 ±29.3	19.9 ±14.9	4.1 ±2.6	NA	54	13.2 ±12.5	6.4 ±5.9	10.9 ±5.9	28.2 ±21.3	28.2 ±21.3	5.9 ±3.3
Complementary therapy cost	88	7.1 ±6.2	5.1 ±4.0	6.7 ±5.5	9.1 ±16.5	14.7 ±47.8	4 ±NA	51	8.1 ±7.5	4.2 ±2.6	4.5 ±7.9	8.5 ±7.9	8.1 ±7.5	NA
*Unweighted total direct non-medical cost*	*(N=320)*	*44.3 ±31.5*	*34.0 ±24.5*	*71.5 ±51.7*	*103.8 ±175.4*	*40.8 ±43.2*	*34.3 ±NA*	*(N=196)*	*55.5 ±67.7*	*35.8 ±16.5*	*67.6 ±53.3*	*56.0 ±43.5*	*33.2 ±20.1*	*28.4 ±18.7*
**No. of complete surveys**	198	41	63	15	33	46	0	111	26	27	10	20	26	2
**Indirect cost,** *Mean* ±SD in USD														
Income lost among those who missed work due to illness	198	39.7 ±35.1	36.1 ±31.4	20.3 ±16.4	59.4 ±70.2	38.0 ±43.8	NA	111	28.1 ±29.5	17.0 ±17.1	37.9 ±27.9	64.3 ±74.2	24.6 ±23.7	6.9 ±1.9
*Unweighted total indirect cost*	*(N=320)*	*25.4 ±33.9*	*28.8 ±31.5*	*6.5 ±13.1*	*34.9 ±61.1*	*23.9 ±39.3*	*NA*	*(N=196)*	*18.3 ±27.2*	*15.3 ±17.0*	*15.8 ±25.9*	*41.5 ±66.8*	*9.4 ±18.9*	*4.6 ±4.2*

Note: * Direct medical costs for Primary Health Centre (PHC) were recorded as a package without itemised components

Only a small proportion of cases were classified as complicated typhoid: 49/891 (5.5%) among children and 56/647 (8.7%) among adults. However, these complicated typhoid cases caused higher direct medical costs than uncomplicated cases. Among children, the mean direct medical costs were US$307.8 ± 214.4 for complicated cases versus US$177.2 ± 133.1 for uncomplicated cases, while the costs in adults were US$316.6 ± 228.2 versus US$200.5 ± 139.5 for complicated and uncomplicated cases, respectively. Complicated cases were mostly treated in tertiary hospitals and private hospitals. The length of stay (LOS) in wards among children with complicated and uncomplicated typhoid cases does not differ significantly, at 3.6 ± 1.3 days and 3.6 ± 1.9 days, respectively. However, LOS is higher in adults with complicated typhoid cases than in those without, at 4.2 ± 2.2 days and 3.5 ± 1.5 days, respectively (Table 5). The mean, median and min-max values of direct medical costs by healthcare facilities and provinces were described for children in S4 Table in S1 File and adults in S5 Table in S1 File.

**Table 5:** Direct medical costs of hospitalised typhoid fever by complication status and age groups.

Parameter	Children (6 m.o. to 17 y.o.)			Adults (≥18 y.o.)		
	No. of non-missing data	Complicated	Uncomplicated	No. of non-missing records	Complicated	Uncomplicated
**No. of records**	891	49	878	647	56	591
**Sample population by facility, n (%)**						
Tertiary public hospital	196 (22.0%)	8 (16.3%)	188 (22.3%)	111 (17.2%)	5 (8.9%)	106 (17.9%)
Secondary public hospital	274 (30.8%)	6 (12.2%)	268 (31.8%)	251 (38.8%)	20 (35.7%)	231 (39.1%)
Private hospital	304 (34.1%)	35 (71.4)	269 (31.9%)	215 (33.3%)	30 (53.6%)	185 (31.3%)
Primary health centre	117 (13.1%)	0 (0.0%)	117 (13.9%)	70 (10.8%)	1(1.8%)	69 (11.7%)
**Direct Medical Cost,** *Mean ± SD in USD*						
Administrative cost	661	7.8 ± 4.6	6.1 ± 5.9	507	5.7 ± 4.8	6.0 ± 5.9
Room cost	771	90.8 ± 94.1	63.5 ± 56.6	573	93.5 ± 90.9	64.1 ± 62.9
Professional fee	771	99.4 ± 70.5	64.4 ± 46.4	572	67.3 ± 48.7	64.9 ± 44.1
Medication cost	771	61.1 ± 69.4	36.4 ± 41.9	576	97.8 ± 126.6	51.9 ± 54.4
Diagnostic cost	733	51.8 ± 49.6	28.1 ± 27.8	512	59.4 ± 44.1	35.5 ± 29.6
*Total direct medical cost*	*878*	*307.8 ± 214.4*	*177.2 ± 133.1*	*567*	*316.6 ± 228.2*	*200.5 ± 139.5*
**Length-of-Stay in wards,** *Mean ± SD in days*	891	3.6 ± 1.3	3.6 ± 1.9	612	4.2 ± 2.2	3.5 ± 1.5

### Direct non-medical and indirect costs

Direct non-medical costs per hospitalised typhoid case were lower than direct medical costs but remained substantial. Among children, the direct non-medical costs ranged from US$30.7 ± 26.2 in PHCs to US$78.2 ± 154.8 in tertiary public hospitals. Among adults, the direct non-medical cost ranged from US$30.9 ± 25.0 in PHCs to US$66.4 ± 86.5 in tertiary public hospitals. The weighted average of direct non-medical out-of-pocket costs incurred totalled US$43.72 ± 16.3 for children and US$39.0 ± 10.9 for adults. This cost was primarily attributed to meals, followed by transportation and food supplements (Figure 2). Among all households with completed self-reported income, the average indirect cost associated with the patient’s/caregiver’s absence from work during the typhoid hospitalisation was US$23.9. ± 5.6 for children and US$17.9 ± 5.5 for adults (Table 3). The distributions of all costs were visualised in Figure 3.

**Fig 2:**
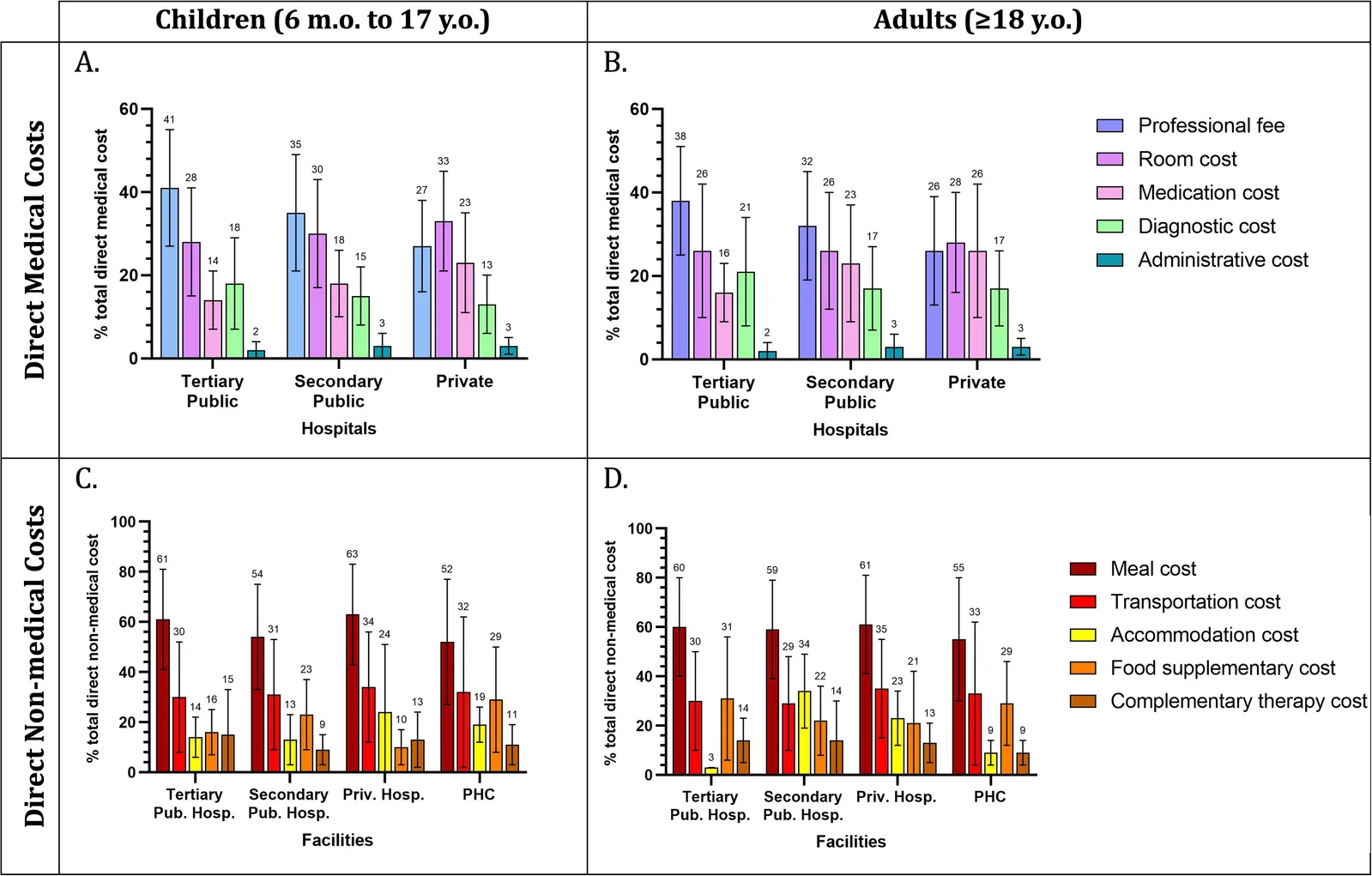
Proportion of total direct medical costs (A-B) and direct non-medical costs (C-D) among child patients (A & C) and adult patients (B & D) hospitalised with typhoid fever, categorised by healthcare facilities.

**Fig 3:**
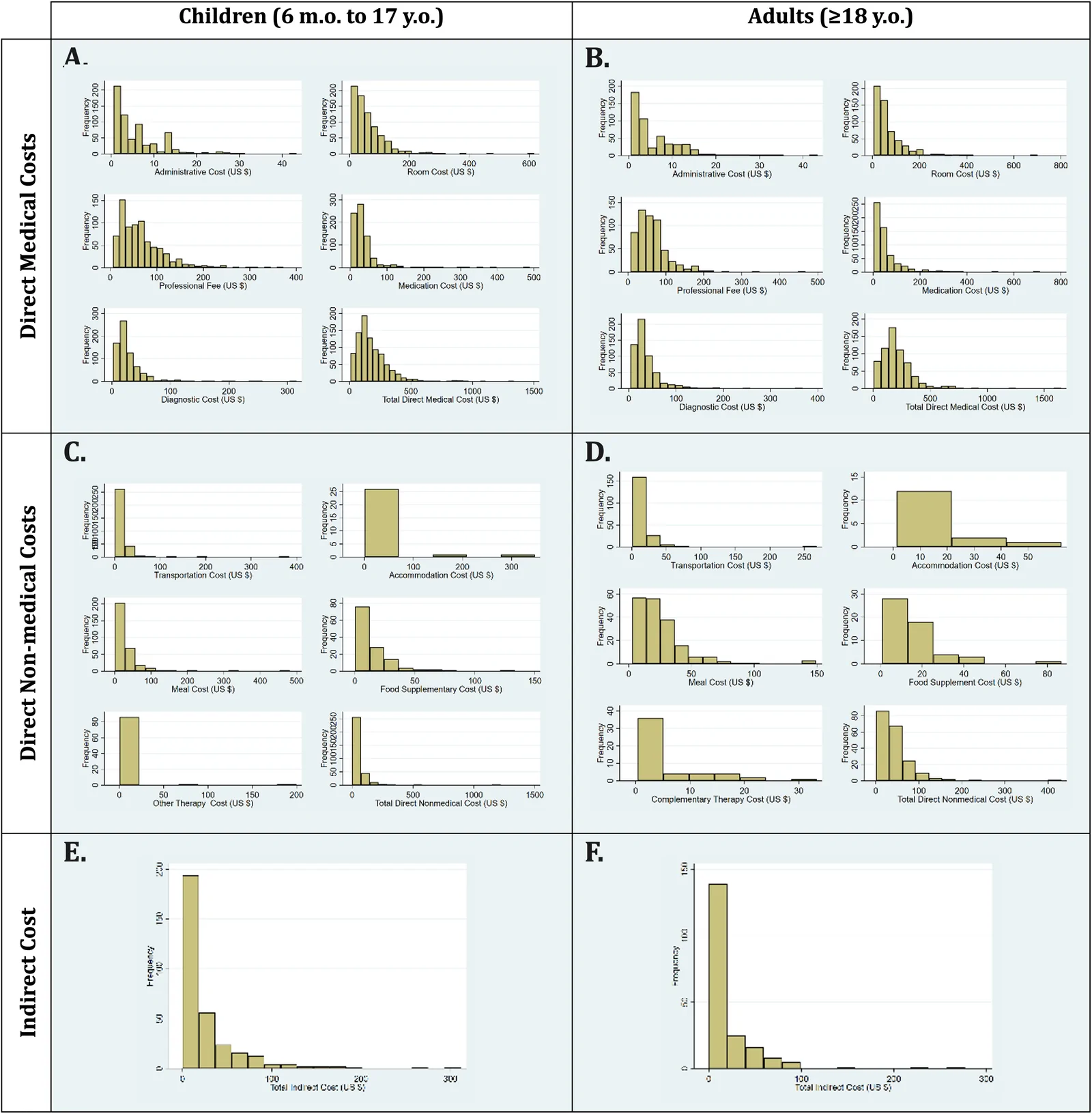
Distribution of direct medical costs (A-B), direct non-medical costs (C-D), and indirect costs (E-F) components among child patients (A, C, E) and adult patients (B, D, F) hospitalised with typhoid fever.

### Sensitivity analysis

Across age groups (children and adults) and healthcare facilities, cost estimates were re-analysed as a sensitivity analysis by excluding the outliers using the Tukey threshold. Sensitivity analyses excluding component-wise outliers showed that cost estimates were robust to extreme values. After outlier exclusion, the weighted mean direct medical cost decreased modestly to US$163.2 ± 88.7 for children and US$174.7 ± 96.7 for adults. Weighted direct non-medical costs decreased to US$38.3 ± 7.3 (children) and US$37.9 ± 6.7 (adults), while weighted indirect costs increased slightly to US$27.5 ± 6.3 (children) and US$22.2 ± 12.5 (adults) (Table 6). The relative differences by facility type and province in the sensitivity analysis remained similar to those in the original main analysis. We also adhered to the consensus-based checklist for cost-of-illness studies to ensure complete reporting (S6 Table in S1 File in the Supplementary Materials) [33].

**Table 6:** Sensitivity analysis of the direct medical cost, direct non-medical cost and indirect cost of hospitalised typhoid fever by healthcare facility type and age groups.

Parameter	Children (6 m.o. to 17 y.o.)					Adults (≥18 y.o.)				
	No. of non-missing records	Public Hospitals		Private Hospitals	Primary Health Centres	No. of non-missing records	Public Hospitals		Private Hospitals	Primary Health Centres
		Tertiary	Secondary				Tertiary	Secondary		
		(Class A/B)	(Class C/D)				(Class A/B)	(Class C/D)		
**No. of complete records**	863	192	274	292	105	619	110	247	195	67
**Care-seeking distribution (weight) across facilities,** *% of all cases*		7.8%	14.0%	49.9%	28.4%		7.8%	14.0%	49.9%	28.4%
**Direct Medical Cost,** *Mean* ± SD in USD										
Administrative cost	654	3.5 ± 3.4	4.6 ± 5. 7	7.5 ± 4.2	NA	498	3.0 ± 3.1	4.5 ± 5.3	7.6 ± 4.0	NA
Room cost	743	49.9 ± 32.7	49.8 ± 39.3	69.1 ± 38.9	NA	556	48.0 ± 44.0	54.1 ± 45.6	71.2 ± 48.1	NA
Professional fee	756	69.9 ± 36.6	54.1 ± 33.3	66.3 ± 46.9	NA	541	60.5 ± 25.7	55.0 ± 31.1	59.2 ± 29.7	NA
Medication cost	771	25.9 ± 32.3	26.9 ±19.9	55.9 ± 59.1	NA	576	27.7 ± 25.5	47.9 ± 48.8	80.9 ± 86.7	NA
Diagnostic cost	733	36.6 ± 46.7	21.7 ± 12.1	32.2 ± 27.2	NA	567	38.1 ± 43.3	29.8 ± 16.9	46.9 ± 36.4	NA
*Unweighted total direct medical cost*	*(N=863)*	*179.2 ± 93.7*	*157.4 ± 86.1*	*227.9 ± 115.2*	47.8 ± 21.8*	*(N=619)*	*170.9 ± 77.3*	*194.9 ± 97.1*	*242.6 ± 89.2*	46.6 ± 28.7*
***Weighted total direct medical cost***		***163.2 ± 88.7***					***174.7 ± 96.7***			
**Direct Non-Medical Cost,** *Mean* ± SD in USD										
Transportation cost	304	12.7 ± 8.7	13.9 ± 10.6	9.4 ± 7.3	6.1 ± 5.4	182	11.5 ± 8.0	11.5 ± 8.4	9.8 ± 8.6	7.0 ± 6.7
Accommodation cost	26	13.5 ± 8.8	17.9 ± 11.9	3.3 ± 2.6	7.8 ± 5.1	15	7.9 ± 7.7	39.7 ± 19.7	8.0 ± 4.5	2.2 ± 0.8
Meal cost	260	23.7 ± 9.4	20.7 ± 10.0	17.5 ± 9.6	14.8 ± 11.2	181	29.7 ± 17.9	25.5 ± 15.5	21.8 ± 14.0	17.7 ± 9.8
Food supplement cost	126	11.9 ± 10.8	17.8 ± 16.0	5.7 ± 5.4	12.9 ± 13.8	53	12 ± 5.6	13.2 ±11.9	13.6 ± 12.7	12.8 ±18.2
Complementary therapy cost	80	5.6 ± 3.7	3.7 ± 2.5	4.4 ± 3.9	4.9 ± 3.4	51	9.9 ±6.9	5.9 ± 7.7	5.0 ± 4.8	2.3 ± 1.1
*Unweighted total direct non-medical Cost*	*(N=306)*	*47.1 ± 26.2*	*53.0 ± 38.6*	*37.2 ± 29.6*	*30.7 ± 26.2*	*(N=192)*	*50.3 ± 29.4*	*45.6 ± 29.6*	*30.7 ± 25.0*	*37.8 ± 27.2*
***Weighted total direct non-medical cost***		***38.3 ± 7.3***					***37.9 ± 6.7***			
**No. of complete surveys**	198	49	57	79	13	111	14	49	34	14
**Indirect cost,** *Mean* ± SD in USD										
Income lost among those who missed work due to illness	198	49.5 ± 60.1	39.4 ± 50.5	46.9 ± 34.4	46.7 ± 42.2	111	21.4 ± 19.4	36.5 ± 26.2	54.3 ± 62.8	27.7 ± 27.0
*Unweighted total indirect cost*	(N=320)**	*33.7 ± 54.6*	*21.8 ± 42.3*	*31.9 ± 35.8*	*20.9 ± 36.4*	(N=196)**	*12.5 ±18.2*	*22.6 ± 27.2*	*18.5 ± 25.6*	*25.7 ± 50.8*
***Weighted total indirect cost***		***27.5 ± 6.3***					***22.2 ± 12.5***			

Note: * Direct medical costs for Primary Health Centre (PHC) were recorded as a package without itemised components.

** Participant counts reflect the analytical sample after missing income values were imputed using the published provincial minimum wage rates [32].

## Discussion

This study examines the direct and indirect expenses incurred for caring for patients hospitalised with probable typhoid fever in Indonesia. Analysing costing data from inpatient cases provides a detailed account of the financial outlays associated with treating hospitalised typhoid fever. Additionally, household surveys provide insights into the socioeconomic impact and potential challenges households face in managing the financial burden of this illness. Understanding these cost dynamics is vital for formulating effective healthcare policies and support systems.

The study revealed that total direct medical expenses for an inpatient case were higher in private hospitals than in public hospitals, consistent with prior research on diarrhea in Indonesia [18]. This difference is likely due to the business’s financial returns, as well as higher unit tariffs for rooms, professional fees, diagnostics, and medical services provided by private hospitals. The cost of inpatient management of typhoid fever were lower in this current study compared with the findings reported in 2002, before the implementation of JKN insurance (total cost of typhoid hospitalisation: 2002: US$884 (children: US$ 616 and adults: US$ 1,095) 2023: $269 (children: US$ 264 and adults: US$ 276) using same 2022 USD currency) [13,14]. Evidence suggests that JKN coverage is associated with lower household out-of-pocket expenditure, including for hospital care, indicating that insurance can affect the distribution of financial payment across BPJS regions, healthcare providers and households [34]. Nevertheless, the present study was not designed to estimate the causal effect of JKN reimbursement on the cost of typhoid fever. Besides, the two studies differ in geographic coverage, study population and participating facilities, case definition, costing methods, clinical guidelines, price year, and health-financing arrangements. Geographically, the previous study was conducted only in the capital province of Jakarta, which does not represent the other provinces in Indonesia [15,18]. Our study covered more provinces, showing the variations of the direct and indirect costs of typhoid hospitalisation across provinces from varied BPJS tariff regions. These estimates provide empirical inputs for further cost-effectiveness and budget-impact analysis of TCV introduction and other public health intervention priorities in Indonesia [12]. For other LMICs, the estimates from this study and further economic evaluation may remain a useful comparator. Still, direct transfer should be undertaken cautiously and should account for the sociodemographical characteristics, disease burden pattern, and other relevant contextual factors [35,36].

Cost-of-illness studies conducted by Surveillance for Enteric Fever in Asia Project (SEAP) II also provided substantial household and healthcare provider costs associated with enteric fever in Bangladesh, Nepal, and Pakistan. The patient and caregiver perspective median costs were approximately US$64 in Bangladesh, US$60 in Nepal, and US$196 in Pakistan in 2018 prices, while provider costs varied by country and facility [37–39]. However, compared with our study, the SEAP II predominantly enrolled blood-culture-confirmed enteric fever, used 2018 prices, and valued provider costs from quantities and unit prices of resources. Therefore, the direct comparison or application with estimates from our study should be made cautiously. Together with other cost evidence across the world, these studies demonstrate that the economic burdens caused by typhoid fever are highly context-dependent and country-specific cost inputs are important for further vaccine economic evaluations [13].

This study shows variations in direct medical and non-medical costs across BPJS regions. Differences in direct medical costs arise from regional tariff systems, which are adjusted according to each region’s economic conditions [16]. In contrast, variations in direct non-medical costs, such as transportation expenses, are likely influenced by the unique geographical characteristics of each area.

A notable strength of this study lies in its comprehensive data collection from diverse healthcare facilities, including public hospitals, private hospitals, and primary health centres. It also encompasses costs borne by households, public insurers, and private insurers. This data was gathered from provinces spanning all regional areas of BPJS (I-V) in Indonesia, covering major islands such as Java, Sumatra, Kalimantan, Sulawesi, and the Nusa Tenggara Islands, to provide an accurate depiction of the nationwide economic burden of typhoid fever.

A limitation was reliance on patient recall and medical records to capture data, which may lead to recall bias and variability in the accuracy of reported expenses. Additionally, the study focuses on hospitalised cases, so it may not capture the economic burden in outpatient facilities or the community, potentially underestimating the overall cost burden of typhoid fever in Indonesia. Furthermore, because provinces and facilities were purposively selected, partly based on their willingness to participate, the study sample cannot be considered probability-based, and this selection approach may have introduced potential selection bias due to systematic differences between participating and non-participating facilities. Another limitation is that a common exchange rate (2023 USD) was applied to facilitate simplicity and consistency; changes in the general price level over the period may have affected the comparability across years. However, for household costs, which were collected over the shorter (2022–2023) period, the potential effect of inflation is expected to be minimal.

An important limitation is that this study includes probable typhoid cases rather than culture-confirmed typhoid cases. Serological tests may produce false-positive or false-negative results, particularly in endemic settings [40,41]. We acknowledge that the costs caused by probable typhoid cases may slightly differ from blood culture-confirmed typhoid costs, especially in terms of duration of stay and disease severity [7]; however, the same tariff may generally be applied to both probable and culture-confirmed typhoid fever cases when they are managed using the same treatment regimens and clinical guidelines within the facility [16]. Additionally, this study did not estimate the cost associated with non-hospitalised typhoid fever cases or costs stratified by antimicrobial resistance. Future studies should prospectively measure costs among outpatient cases, non-medical care, chronic carier, and patients with antimicrobial resistance. Furthermore, this study did not collect the productivity loss due to premature death and outside the hospitalisation period, which might underestimate the indirect cost.

This study’s findings further highlight the potential cost savings and cost-effectiveness of implementing preventive strategies, including the introduction of typhoid conjugate vaccines (TCV) into Indonesia’s National Immunisation Program (NIP), with potential relevance to many other LMICs. In 2026, UNICEF’s TCV procurement prices were approximately US$0.99–1.34 per dose for Gavi-eligible countries and US$1.25–1.50 per dose for middle-income countries under pricing arrangements, depending on manufacturers and product [42]. Furthermore, Indonesia’s state-owned vaccine manufacturer, PT Bio Farma, is expected to produce TCV locally, following regulatory approval from the Indonesian Food and Drug Authority in 2023 [43]. The availability of locally manufactured TCV could further support vaccine accessibility and potentially reduce the costs associated with introducing TCV in Indonesia. By reducing the incidence of severe typhoid fever that requires hospitalisation, the vaccination could alleviate the financial burden on households and healthcare systems [7]. Accordingly, we have undertaken a cost-effectiveness and budget impact analysis of TCV using cost data generated from this study to inform TCV vaccine introduction in Indonesia [12].

## Conclusion

Hospitalised typhoid fever imposes a substantial economic burden on Indonesia’s health system for both children and adults. These estimates of direct and indirect costs, generated across diverse healthcare settings and provinces, can support projections of potential healthcare costs and inform future cost-effectiveness analyses of introducing typhoid conjugate vaccines into Indonesia’s National Immunisation Program.

## Supporting information

S1 File Supplementary Materials. (PDF)
