# Supplementary material for: Estimating direct and indirect costs of hospitalised typhoid fever in Indonesia: A multisite healthcare facility and community survey
Source: PLoS One. 2026 Oct 26;21(10):e0360948. doi: 10.1371/journal.pone.0360948 (PMC13704798; doi:10.1371/journal.pone.0360948)
Supplement: Supplementary file 1 [file pone.0360948.s001.pdf]

Supplementary Materials

Estimating direct and indirect costs of hospitalised typhoid fever in Indonesia: a multisite healthcare facility and community survey

Jarir At Thobari [1,2*], Ida Safitri Laksono [1,3*], Jonathan Hasian Haposan [1,4,5,6], Hardini Tri Indarti [1,7], Asal Wahyuni Erlin Mulyadi [1,8], Alindina Anjani [1,9], Emma Watts [4], Julie Bines [4,6,10]

[a] Center for Child Health, Faculty of Medicine, Public Health and Nursing, Universitas Gadjah Mada, Yogyakarta, Indonesia

[b] Department of Pharmacology and Therapy, Faculty of Medicine, Public Health and Nursing, Universitas Gadjah Mada, Yogyakarta, Indonesia

[c] Department of Child Health, Faculty of Medicine, Public Health and Nursing, Universitas Gadjah Mada, Yogyakarta, Indonesia and Dr. Sardjito General Hospital, Yogyakarta, Indonesia

[d] Enteric Diseases Research Group, Murdoch Children's Research Institute, Parkville, Australia

[e] Department of Biostatistics, Epidemiology, and Population Health, Faculty of Medicine, Public Health and Nursing, Universitas Gadjah Mada, Yogyakarta, Indonesia

[f] Department of Paediatrics, the University of Melbourne, Parkville, Australia

[g] STIKes Budi Luhur Cimahi, West Java, Indonesia

[h] Faculty of Social and Political Sciences, Universitas Sebelas Maret Surakarta, Central Java, Indonesia

[i] Department of Internal Medicine, Faculty of Medicine, Public Health and Nursing, Universitas Gadjah Mada, Yogyakarta, Indonesia

[j] Department of Gastroenterology and Clinical Nutrition, the Royal Children's Hospital, Parkville, Australia

Corresponding author:

1. Dr. Ida Safitri Laksono, MD (Consultant Peadiatric Infectious Diseases) (idalaksono@ugm.ac.id)
2. Prof. Jarir At Thobari, MD, PhD, Dpharm (j.atthobari@ugm.ac.id)

Table S1. Allocation of retrospective record collection by facility type, province, and age groups

No	Province	Type	Children	Adults
			**Retrospective records, n (%)**	**Retrospective records, n (%)**
	West Java		*99 (11.11)*	*60 (9.27)*
1	West Java	Primary Health Centre	24 (2.69)	15 (2.32)
2	West Java	Private Hospital	22 (2.47)	15 (2.32)
3	West Java	Secondary Public Hospital	33 (3.70)	15 (2.32)
4	West Java	Tertiary Public Hospital	20 (2.24)	15 (2.32)
	Central Java		*255 (28.62)*	*162 (25.04)*
5	Central Java	Primary Health Centre	18 (2.02)	10 (1.55)
6	Central Java	Primary Health Centre	60 (6.73)	45 (6.96)
7	Central Java	Private Hospital	93 (10.44)	36 (5.56)
8	Central Java	Secondary Public Hospital	44 (4.94)	41 (6.34)
9	Central Java	Tertiary Public Hospital	40 (4.49)	30 (4.64)
	South Sumatra		*128 (14.37)*	*109 (16.85)*
10	South Sumatra	Private Hospital	62 (6.96)	60 (9.27)
11	South Sumatra	Secondary Public Hospital	5 (0.56)	3 (0.46)
12	South Sumatra	Tertiary Public Hospital	61 (6.85)	46 (7.11)
	South Sulawesi		*210 (23.57)*	*184 (28.44)*
13	South Sulawesi	Primary Health Centre	51 (5.72)	46 (7.11)
14	South Sulawesi	Private Hospital	74 (8.31)	68 (10.51)
15	South Sulawesi	Secondary Public Hospital	40 (4.49)	30 (4.64)
16	South Sulawesi	Tertiary Public Hospital	45 (5.05)	40 (6.18)
	South Kalimantan		*104 (11.67)*	*78 (12.06)*
17	South Kalimantan	Primary Health Centre	3 (0.34)	21 (3.25)
18	South Kalimantan	Private Hospital	36 (4.04)	21 (3.25)
19	South Kalimantan	Secondary Public Hospital	30 (3.37)	18 (2.78)
20	South Kalimantan	Tertiary Public Hospital	35 (3.93)	18 (2.78)
	East Nusa Tenggara		*95 (10.66)*	*54 (8.35)*
21	East Nusa Tenggara	Private Hospital	27 (3.03)	15 (2.32)
22	East Nusa Tenggara	Secondary Public Hospital	48 (5.39)	32 (4.95)
23	East Nusa Tenggara	Tertiary Public Hospital	20 (2.24)	7 (1.08)
	**Total Records**		**891 (100)**	**647 (100)**

Table S2. Allocation of prospective community surveys by inpatient facility type, province, and age groups

No	Province	Type	Children	Adults
			**Prospective surveys, n (%)**	**Prospective surveys, n (%)**
	Central Java		*83 (25.23)*	*30 (14.85)*
1	Central Java	Primary Health Centre	9 (2.74)	9 (4.46)
2	Central Java	Private Hospital	53 (16.11)	6 (2.97)
3	Central Java	Secondary Public Hospital	11 (3.34)	12 (5.94)
4	Central Java	Tertiary Public Hospital	10 (3.04)	3 (1.49)
	West Java		*66 (20.06)*	*42 (20.79)*
5	West Java	Primary Health Centre	9 (2.74)	5 (2.48)
6	West Java	Private Hospital	20 (6.08)	15 (7.43)
7	West Java	Secondary Public Hospital	21 (6.38)	16 (7.92)
8	West Java	Tertiary Public Hospital	16 (4.86)	6 (2.97)
	South Sumatra		*47 (14.29)*	*25 (12.38)*
9	South Sumatra	Primary Health Centre	0 (0.00)	0 (0.00)
10	South Sumatra	Private Hospital	3 (0.91)	2 (0.99)
11	South Sumatra	Secondary Public Hospital	32 (9.73)	21 (10.40)
12	South Sumatra	Tertiary Public Hospital	12 (3.65)	2 (0.99)
	South Sulawesi		*59 (17.93)*	*32 (15.84)*
13	South Sulawesi	Primary Health Centre	5 (1.52)	5 (2.48)
14	South Sulawesi	Private Hospital	23 (6.99)	15 (7.43)
15	South Sulawesi	Secondary Public Hospital	20 (6.08)	10 (4.95)
16	South Sulawesi	Tertiary Public Hospital	11 (3.34)	1 (0.50)
	South Kalimantan		*73 (22.19)*	*69 (34.16)*
17	South Kalimantan	Primary Health Centre	6 (1.82)	2 (0.99)
18	South Kalimantan	Private Hospital	26 (7.90)	36 (17.82)
19	South Kalimantan	Secondary Public Hospital	18 (5.47)	18 (8.91)
20	South Kalimantan	Tertiary Public Hospital	23 (6.99)	13 (6.44)
	East Nusa Tenggara		*1 (0.30)*	*4 (1.98)*
21	East Nusa Tenggara	Primary Health Centre	0 (0.00)	0 (0.00)
22	East Nusa Tenggara	Private Hospital	0 (0.00)	0 (0.00)
23	East Nusa Tenggara	Secondary Public Hospital	1 (0.30)	4 (1.98)
24	East Nusa Tenggara	Tertiary Public Hospital	0 (0.00)	0 (0.00)
	**Total Surveys**		**329 (100)**	**202 (100)**

Table S3. Serodiagnostic laboratory test results of retrospective records

**Serodiagnostic Laboratory Test Results**	**Children (N=891)**		**Adults (N = 647)**	
	n	*(% of N)*	*n*	*(% of N)*
Positive Widal test only	614	(68.9)	478	(73.9)
Positive TUBEX TF only	124	(13.9)	70	(10.8)
Positive Typhoid IgM antibody test only	86	(9.7)	78	(12.1)
Positive Typhidot only	17	(1.9)	6	(0.9)
At least 2 tests positive	50	(5.6)	15	(2.3)

Note: Serodiagnostic testing practices, including standard operating procedures and the availability of laboratory tests, varied across healthcare facilities and sites. Therefore, the distribution presented in this table reflects the tests performed and recorded at participating facilities and should not be interpreted as representing epidemiologic distribution of typhoid based on serodiagnostic test results. All prospective participants were tested for Widal and/or Typhidot tests before the inclusion to the study.

Table S4. Direct medical cost of hospitalised typhoid fever based on healthcare facility types and provinces (children)

	**West Java**	**Central Java**	**South Sumatra**	**South Sulawesi**	**South Kalimantan**	**East Nusa Tenggara**
	**Region I**	**Region I**	**Region II**	**Region III**	**Region IV**	**Region V**
No. of patients	181	229	153	127	160	28
Healthcare Type:						
Tertiary (Class A/B) Public Hospital, n	36	30	35	27	47	20
Mean, in US$	177.04	113.93	216.91	362.94	134.78	150.8
Median, in US$	172.4	100.81	193.68	331.37	129.52	141.73
Min – Max, in US$	57.00-336.36	45.73-196.56	94.06-576.16	134.91-791.19	69.19-208.60	84.49-295.88
Secondary (Class C/D) Public Hospital, n	61	51	74	40	45	3
Mean, in US$	74.83	127.89	243.77	202.9	118.69	179.71
Median, in US$	67.45	114.79	237.19	207.3	109.84	149.59
Min – Max, in US$	25.66-199.60	70.28-281.87	115.57-495.44	89.80-320.65	65.96-231.52	105.87-283.72
Private Hospital, n	60	93	44	40	62	5
Mean, in US$	425.03	140.93	177.27	332.55	265.76	229.3
Median, in US$	341.36	130.42	144.47	309.65	239.55	180.79
Min – Max, in US$	172.00-1336.247	74.04-367.32	69.57-452.65	136.78-864.62	81.95-1095.09	78.60-442.15
Primary Health Centre, n	24	55	0	20	6	0
Mean, in US$	45.16	43.97	NA	60.38	51.11	NA
Median, in US$	53.33	42.07	NA	46.67	40	NA
Min – Max, in US$	6.67-98.66	23.33	NA	11.67-139.20	26.67-106.67	NA

Table S5. Direct medical cost of hospitalised typhoid fever based on healthcare facility types and provinces (adult)

	**West Java**	**Central Java**	**South Sumatra**	**South Sulawesi**	**South Kalimantan**	**East Nusa Tenggara**
	**Region I**	**Region I**	**Region II**	**Region III**	**Region IV**	**Region V**
No of patients	127	130	127	90	139	31
Healthcare Type:						
Tertiary (Class A/B) Public Hospital, n	21	18	18	15	32	7
Mean, in US$	193.84	176.33	170.89	151.12	141.95	364.05
Median, in US$	182.67	148.42	189.61	120.58	138.14	299.62
Min – Max, in US$	106.99 - 341.13	84.82 - 314.66	26.98 – 257.93	18.16-490.71	65.15 - 235.68	122.03 - 873.21
Secondary (Class C/D) Public Hospital, n	46	46	68	30	40	21
Mean, in US$	124.94	129.37	276.11	255.36	172.62	257.61
Median, in US$	116.36	115.39	253.4	219.48	166.13	274.67
Min – Max, in US$	42.53 - 246.99	40.17-371.46	96.87-597.39	83.63 - 609.55	88.23 – 561.76	151.29 - 437.98
Private Hospital, n	45	36	41	30	60	3
Mean, in US$	469.26	304.77	204.3	239.68	220.1	505.2
Median, in US$	357.19	287.24	199.82	218.72	196.28	169.05
Min – Max, in US$	194.36 – 1647.20	108.24-713.16	80.22-371.27	118.23-461.87	73.83 – 615.62	98.53 - 1248.02
Primary Health Centre, n	15	30	0	15	7	0
Mean	3068	62.17	NA	38.63	31.32	NA
Median	30.33	53.33	NA	35	26.67	NA
Min – Max	3.33-66.67	28.73 – 173.43	NA	8.28 – 66.67	26.97-53.33	NA

Table S6. The consensus-based checklist for cost-of-illness studies [1]

Item	Question	Answer[a]	Supportive information
**Study characteristics**			
Question/objective	Is a well-defined research question or objective stated?	Yes	Introduction
Population	Is the study population described?	Yes	Materials and methods
Perspective	a) Is (are) the chosen study perspective(s) stated?	Yes	Materials and methods
	b) If so, is (are) the chosen study perspective(s) justified?	Yes	Materials and methods
**Methodology and cost analysis**			
Epidemiological approach	Is the epidemiological approach reported (e.g., prevalence, incidence)?	Incidence	Materials and methods
Costing approach	Is the costing approach reported (e.g., top-down, bottom-up)?	Bottom-up	Materials and methods
Data collection approach	Is the data collection process reported (e.g., prospective, retrospective)?	Prospective & Retrospective	Materials and methods
Identification	a) Are all components of resource use identified that are relevant to the condition/disease, population, intervention, study objectives, and study perspective?	Yes	Materials and methods
	b) If not, is a justification provided for excluding relevant components of resource use?		
Measurement	a) Are all included components of resource use measured?	Yes	Materials and methods
	b) If not, is a justification provided for not measuring certain components of resource use?		
Valuation	a) Are all included components of resource use valued in monetary terms?	Yes	Materials and methods
	b) If not, is a justification provided for not valuing certain components of resource use?		
Time horizon	a) Is the chosen time horizon specified?	Yes	Materials and methods
	b) If so, is the chosen time horizon justified?		
Discounting	a) Are future costs discounted?	N/A	
	b) If so, is a justification provided for the discount rate?		
Sensitivity	a) Are all variables whose values are uncertain subjected to sensitivity analysis?	Yes	Materials and methods
	b) If so, is a justification provided for which variables are subjected to sensitivity analysis?		

	c) Are analyses done on relevant subgroups?	Yes	Materials and methods
**Results and reporting**			
Cost sectors	Are the study results presented transparently by cost category/sector?	Yes	Results
Generalizability	Do the authors discuss the generalizability of study results (e.g., comparing the results to other patient/client groups or/in other settings)?	Yes	Results
Limitations	Do the authors discuss important limitations?	Yes	Discussion
Ethical and distributional issues	a) Do the authors discuss ethical issues?	N/A	
	b) Do the authors discuss distributional issues?	Yes	Discussion
Conflict of interest	Do the authors report any potential conflicts of interest?	Yes	Disclosure Statement

[a] Answer categories: Yes, No, Partially, Not Applicable (N/A), and Unclear.

References:

1. Schnitzler L, Roberts TE, Jackson LJ, Paulus AT, Evers SM. A consensus-based checklist for the critical appraisal of cost-of-illness (COI) studies. International journal of technology assessment in health care. 2023 Jan;39(1):e34.
